# Hierarchical clustering of prolonged post-concussive symptoms after 12 months: symptom-centric analysis and association with functional impairments

**DOI:** 10.1080/02699052.2022.2158229

**Published:** 2022-12-18

**Authors:** Ali Alim-Marvasti, Narayan Kuleindiren, Federico Tiersen, Monika Johal, Aaron Lin, Hamzah Selim, Raphael Rifkin-Zybutz, Mohammad Mahmud

**Affiliations:** a Research Division, Mindset Technologies Ltd, London, UK; b Wellcome/EPSRC Centre for Interventional and Surgical Sciences (WEISS), University College London, London, UK; c Department of Medical Physics and Biomedical Engineering, University College London, London, UK; d Wellcome/EPSRC Centre for Interventional and Surgical Sciences (WEISS), London, UK; e School of Medicine, Imperial College London, London, UK; f University of Birmingham Medical School, Birmingham, UK; g Department of Brain Sciences, Imperial College London, London, UK

**Keywords:** Rivermead, traumatic brain injury, machine learning, principal components analysis, factor analysis, Post-concussion syndrome

## Abstract

**Background:**

Following a concussion, approximately 15% of individuals experience persistent symptoms that can lead to functional deficits. However, underlying symptom-clusters that persist beyond 12 months have not been adequately characterized, and their relevance to functional deficits are unclear. The aim of this study was to characterize the underlying clusters of prolonged post-concussive symptoms lasting more than 12 months, and to investigate their association with functional impairments.

**Methods:**

Although hierarchical clustering is ideally suited in evaluating subjective symptom severities, it has not been applied to the Rivermead Post-Concussion Questionnaire (RPQ). The RPQ and functional impairments questions were administered via a smartphone application to 445 individuals who self-reported prolonged post-concussive symptoms. Symptom-clusters were obtained using agglomerative hierarchical clustering, and their association with functional deficits were investigated with sensitivity analyses, and corrected for multiple comparisons.

**Results:**

Five symptom-clusters were identified: headache-related, sensitivity to light and sound, cognitive, mood-related, and sleep-fatigue. Individuals with more severe RPQ symptoms were more likely to report functional deficits (*p* < 0.0001). Whereas the headache and sensitivity clusters were associated with at most one impairment, at-least-mild sleeping difficulties and fatigue were associated with four, and moderate-to-severe cognitive difficulties with five (all *p* < 0.01).

**Conclusions:**

Symptom-clusters may be clinically useful for functional outcome stratification for targeted rehabilitation therapies. Further studies are required to replicate these findings in other cohorts and questionnaires, and to ascertain the effects of symptomatic intervention on functional outcomes.

## 1. Introduction

Concussion is considered a mild subset of traumatic brain injury (TBI) making up around 80% of all TBI (1). The incidence of concussion is estimated to be between 0.1% to over 1% of the general population (1,2), and it is a major cause of morbidity following domestic accidents, high-impact sports (3), and in military veterans (4,5). About half of the one million people with TBI who attend emergency departments in the United Kingdom annually experience post-concussive symptoms (6–8). Concussive symptoms include headaches, dizziness, nausea, impaired attention and memory, changes in sleep, and depression or anxiety (9).

Although most concussive symptoms resolve within 3 months (10), a minority of up to 15% of individuals experience persistent post-concussive symptoms lasting more than 3 months, or even more than 12 months, sometimes without structural imaging correlates (11,12). Persistent post-concussive symptoms are associated with chronic morbidity, lower quality of life (13), and functional deficits including reduced ability to work (14), and loss of social autonomy (15). Therefore, despite progress with modern imaging modalities and biomarkers (16), clinical symptoms remain imperative for patient-centered evaluations in identifying potentially treatable long-term symptoms.

However, the precise role of different post-concussive symptomatology in bringing about different functional deficits has not been adequately investigated, partly due to the use of inappropriate statistical techniques for the evaluation of post-concussive symptom questionnaires (9).

Post-concussion symptoms can be assessed using self-reported questionnaires that evaluate the presence of, or change in, symptoms, their frequency and severity, and associated functional impairments (17). The 16-item Rivermead Post Concussion Symptoms Questionnaire (RPQ-16) measures the severity of symptoms with good test–retest reliability (18). Although functional impairments are not part of the questionnaire, RPQ-16 was originally intended to help assess the degree to which symptoms may contribute to psychosocial dysfunction (18).

Previous studies have investigated the underlying symptom-structure of the RPQ-16 using principal components (PCA) and confirmatory factor analyses (CFA) (9). CFA and PCA make assumptions of linearity that are problematic when applied to self-reported symptom questionnaires such as the RPQ. Despite these methods not being the most suited for ordinal scales (9), their application in symptomatic profiling of TBI has not been restricted to RPQ, having also been applied to the 22 question Post-Concussion Symptom Scale (19). Even though CFA and PCA are distinct methods that often produce similar results when applied to the same dataset (20), their applications to various RPQ-16 datasets have yielded different results, including three and four factor structures (11,21,22), with some suggesting dynamic changes in symptom structures within the first year after injury, while other studies have contradicted this (23,24). Besides differences in cohorts, there may be other explanations for these inconsistent findings, including incongruence amongst, and between, physicians and patients in their interpretations of symptoms. For example, a clinician may interpret “double vision” as diplopia whereas patients may perceive it to adequately describe blurred vision. As another example, dizziness could mean vertigo at rest (vestibular) for one patient, or motion sensitivity from migraine, or benign paroxysmal positional vertigo (BPPV) for another. Such diverging interpretations have implications for symptom-groupings due to errors in clustering different symptoms under single nonspecific terminologies. Additionally, an individual’s interpretation of symptoms may change with time. For example, an individual with acute vertigo from BPPV may report “dizziness” (question 2 of RPQ-16) and although later resolved, may still report “dizziness” to be present due to chronic migraines (25). Unlike PCA and CFA, hierarchical clustering does not assume linearity and does not impose theoretical understandings of underlying factor structures to subjective severity scores. Therefore, compared to PCA and CFA, hierarchical clustering may be a more suitable method to evaluate subjectively scored symptoms (9,26).

The aim of this study was to perform a symptom-centric analysis of prolonged symptoms following a concussion. The primary purpose was to characterize the underlying symptom-structure of prolonged post concussive symptoms that last more than 12 months using agglomerative hierarchical clustering on responses to the RPQ, and to compare the symptom-clusters obtained to the symptom factors obtained from previous methods. Although the majority of post-concussive symptoms usually resolve within the first few months, the persistence of symptoms causes significant morbidity (11,12). Therefore, finding unique clinical profiles in individuals with notably prolonged symptoms could be valuable in stratifying concussion outcomes, supplementing interventions that have been shown to reduce morbidity in randomized control trials (27). Therefore, the secondary aim of this study was to investigate if these symptom-clusters were then associated with unique functional impairments, which were captured along with the RPQ-16 responses using a smartphone application and a bespoke abbreviated functional impairments questionnaire suitable for remote self-reporting.

## 2. Methods

### 2.1. Setting: smartphone application

The *Mindstep (Mindset4Dementia)* application consisted of a 5-minute questionnaire where information on common risk factors for cognitive impairment were gathered, including concussion. The application routinely asked about a history of concussion and, if present, participants were automatically shown the RPQ-16. The application is freely available to iPhone users in the Apple Application Store and has been previously validated (28).

### 2.2. Participants

Participants were individuals over the age of 18, who of their own accord, downloaded and self-reported a prior diagnosed concussion within the *Mindstep* (*Mindset4Dementia)* application. Eligible participants were not sought out nor paid. Participants gave informed consent for the collection and use of anonymized data for predictive analytics, as described previously (28,29). Aconvenience sample of consecutive data was obtained between the 1^st^ of March to the 14^th^ of April 2021 from individuals who replied in the affirmative to the following question:

“Have you ever had a diagnosed concussion?”

Participants were dichotomized into two groups based on interval since concussion. “Prolonged” symptoms were defined as those persisting for 12 months or more after a concussion, and “early” symptoms as those occurring within 12 months.

The term “prolonged” (≥12 months) was used to distinguish this from the usual definition of “persistent” post-concussion syndrome (>3 months) (10). This longer period of 12 months also ensured that more stable symptoms were captured as post-concussive symptoms may change within the first year (23), and longer symptoms are more likely to give meaningful stable associations with functional outcomes (15).

Although no data was available on the severity of concussion as this was self-reported, the majority of cases are likely to have fallen into the *symptomatic (possible)* or *mild (probable)* TBI categories as defined by the Mayo system (30,31).

Participants were therefore a convenience sample with the majority reporting (prolonged) post-concussion symptoms and were likely worried about cognitive repercussions.

### 2.3. Data

The application routinely collected data on participants’ ages, presence of comorbidities, RPQ-16 symptoms (if the user gave a history of concussion), duration since concussion, and self-reported functional impairments. These data, and other data that were collected as part of the app, such as smoking status and alcohol consumption, are shown for the prolonged group in Table 1, and for the “early” group (less than 12 months) in **Supplementary Table 1**. Data was fully anonymized and stored in secure Amazon web services cloud. The full range of other data that were routinely collected by the application but were not the focus of this study and therefore not analyzed, have been previously described (28,29).

**Table 1.** Demographics and Comorbidities of Prolonged Symptoms Group.

Prolonged Symptoms ≥ 12 months (n = 445)	
Age = 47.2 ± 12.9 years (mean, std)	
Variable	Occurrence (%)
RPQ-16 Total Score ≥ 16	153 (34.4%)
Covid-19 Positive	57 (12.8%)
Prediabetes	13 (2.9%)
Diabetes	24 (5.4%)
Hypercholesterolaemia	43 (9.7%)
Hypertension	81 (18.2%)
Dementia	3 (0.7%)
Family History of Dementia	172 (38.7%)
Migraine	15 (3.4%)
Parkinsons	1 (0.2%)
Stroke	2 (0.4%)
Epilepsy	4 (0.9%)
Anxiety	33 (7.4%)
Depression	39 (8.8%)
Antidepressants	35 (7.9%)
PTSD	9 (2.0%)
Bipolar	5 (2.1%)
Carer	76 (17.1%)
Quit Alcohol	76 (17.1%)
Current Smoker	58 (13.0%)
Ex-Smoker	123 (27.6%)
2 to 4 Concussions	183 (41.1%)
4 or more Concussions	81 (18.2%)
Alcohol >14units	32 (7.2%)
**Functional Impairments (n = 240)**	
Appointments	83 (34.6%)
Conversations	108 (45.0%)
Driving or Cycling	15 (6.2%)
Mental Math	66 (27.5%)
Paperwork	87 (36.2%)
Planning	69 (28.7%)
Other	10 (4.2%)

*Comorbidities and basic demographics for the prolonged group. PTSD = Post Traumatic Stress Disorder.*

**Table 2.** Summary of Self-Reported Functional Impairments.

Self-reported functional deficit	Frequency (n, %) Total data available: N = 254
Remembering Appointments	87 (34.3%)
Difficulty with Conversations	114 (44.9%)
Difficulty Driving or Cycling	15 (5.9%)
Mental Math	70 (27.6%)
Paperwork	94 (37.0%)
Planning Ahead	74 (29.1%)
Other Deficit(s)	10 (3.9%)
None	49 (19.2%)

*Functional impairments. Data for all 254 participants who completed the functional deficits questionnaire are summarized.*

**Table 3.** Analysis by Mild Symptom-Clusters in Prolonged Symptoms Group.

		Severity at least mild		
RPQ-16 Symptom-Cluster	Functional Impairments	Mean Severity of symptoms within the cluster (Main Analysis) (Bonferroni p < 0.05/30) OR [95% CI]	Sensitivity Analysis: At least one symptom from the cluster	Sensitivity Analysis: All symptoms from the cluster
**Headache-related**	Appointments	NS*	-	-
	Conversations	NS*	-	-
	Driving	NS	-	-
	Math	NS*	-	-
	Paperwork	NS	-	-
	Planning	NS	-	-
**Sensitivity**	Appointments	NS*	-	-
	Conversations	NS*	-	-
	Driving	NS	-	-
	Math	2.98 [1.63, 5.45]	2.44 [1.36, 4.36]	2.72 [1.41, 5.25]
	Paperwork	NS	-	-
	Planning	NS	-	-
**Sleep-Fatigue**	Appointments	2.44 [1.41, 4.22]	2.83 [1.54, 5.21]	2.81 [1.62, 4.86]
	Conversations	2.7 [1.6, 4.56]	2.71 [1.55, 4.74]	3.48 [2.04, 5.94]
	Driving	NS	-	-
	Math	2.64 [1.46, 4.78]	1.75 [0.94, 3.26]	2.17 [1.22, 3.85]
	Paperwork	2.79 [1.61, 4.82]	2.38 [1.33, 4.28]	2.23 [1.31, 3.82]
	Planning	NS*	-	-
**Cognitive**	Appointments	2.93 [1.61, 5.34]	3.56 [1.78, 7.11]	2.72 [1.57, 4.71]
	Conversations	4.41 [2.47, 7.88]	4.57 [2.40, 8.71]	3.77 [2.21, 6.46]
	Driving	NS	-	-
	Math	NS	-	-
	Paperwork	NS*	-	-
	Planning	NS*	-	-
**Emotional**	Appointments	NS*	-	-
	Conversations	3.05 [1.79, 5.18]	2.87 [1.59, 5.17]	2.71 [1.48, 4.97]
	Driving	NS	-	-
	Math	NS	-	-
	Paperwork	2.57 [1.49, 4.44]	2.79 [1.48, 5.24]	2.37 [1.30, 4.30]
	Planning	NS*	-	-

*Presence of at least mild symptoms for membership of clusters. Sensitivity analyses showed effect sizes between cluster-membership and functional impairments to be relatively robust to the choice of membership criteria, except for sleep-fatigue cluster and mental math, where having at least one symptom resulted in an insignificant odds ratio.*

NS = not significant after Bonferroni correction. * = significant without Bonferroni correction p < 0.05.

**Table 4.** Analysis by Moderate Symptom-Clusters in Prolonged Symptoms Group.

		Severity at least moderate		
RPQ-16 Symptom-Cluster	Functional Impairments	Mean Severity of symptoms within the cluster (Main Analysis) (Bonferroni p < 0.05/30) OR [95% CI]	Sensitivity Analysis: At least one symptom from the cluster	Sensitivity Analysis: All symptoms from the cluster
**Headache-related**	Appointments	NS	-	-
	Conversations	NS	-	-
	Driving	NS*	-	-
	Math	NS	-	-
	Paperwork	NS	-	-
	Planning	NS	-	-
**Sensitivity**	Appointments	NS	-	-
	Conversations	NS	-	-
	Driving	NS	-	-
	Math	NS*	-	-
	Paperwork	NS	-	-
	Planning	NS	-	-
**Sleep-Fatigue**	Appointments	2.76 [1.58, 4.83]	2.28 [1.32, 3.93]	2.47 [1.35, 4.55]
	Conversations	3.71 [2.12, 6.52]	2.67 [1.57, 4.53]	3.58 [1.90, 6.74]
	Driving	NS	-	-
	Math	NS*	-	-
	Paperwork	NS*	-	-
	Planning	NS*	-	-
**Cognitive**	Appointments	3.79 [2.15, 6.70]	3.35 [1.92, 5.85]	4.37 [2.25,8.50]
	Conversations	3.75 [2.13, 6.60]	4.79 [2.73, 8.39]	3.87 [1.95, 7.70]
	Driving	NS	-	-
	Math	2.70 [1.50, 4.86]	2.60 [1.45, 4.64]	3.19 [1.65, 6.17]
	Paperwork	2.82 [1.62, 4.93]	1.98 [1.15, 3.40]	2.28 [1.20, 4.33]
	Planning	3.15 [1.76, 5.64]	2.73 [1.54, 4.85]	3.27 [1.69, 6.30]
**Emotional**	Appointments	2.75 [1.50, 5.02]	2.91 [1.68, 5.04]	1.35 [0.50, 3.70]
	Conversations	3.17 [1.71, 5.87]	3.46 [2.02, 5.92]	2.38 [0.85, 6.67]
	Driving	NS	-	-
	Math	NS*	-	-
	Paperwork	2.05 [1.13, 3.73]	2.70 [1.57, 4.65]	2.09 [0.78, 5.64]
	Planning	NS	-	-

*Presence of moderate-to-severe symptoms for cluster membership. Sensitivity analyses showed effect sizes between cluster-membership and functional impairments to be relatively robust to the choice of membership criteria, except for the emotional-cluster, where having all symptoms from the cluster resulted in all statistically significant results to become insignificant.*

NS = not significant after Bonferroni correction. * = significant without Bonferroni correction p < 0.05.

**Table 5:** Analysis by Functional Impairments in Prolonged Symptoms Group

**Functional Impairment**	**Total RPQ-16 score**	**Migraines**	**Sensitivity**	**Sleep**	**Cognitive**	**Emotional**
Appointments	*p<*0.001	NS*	NS	NS*	NS*	NS*
Conversations	*p*<0.0001	NS*	NS	NS*	*p<*0.001	NS*
Driving	NS	NS	NS	NS	NS	NS
Maths	NS*	NS	NS*	NS	NS	NS
Paperwork	NS*	NS	NS	NS	NS	NS*
Planning	NS	NS	NS	NS	NS	NS

*Analysis by functional impairments. The first column compares the severity of total RPQ-16 scores with and without the functional impairment. The rest of the columns compare the severity of mean of each symptom-cluster with and without the functional impairment.*

NS = not significant after Bonferroni correction. * = significant without Bonferroni correction p < 0.05.

#### 2.2.1. RPQ-16 symptom scores

RPQ-16 consists of rating 16 symptoms compared to pre-injury levels using a five-point Likert-like scale from “0” through to “4” (18). The questionnaire asks participants to compare their current symptoms to those before the head injury and rate them as “not experienced at all,” “present but no more of a problem,” “mild problem,” “moderate problem,” or “severe problem.” All 16 questions from the questionnaire were presented, summarized in **Supplementary Table 2.**

As in previous studies, RPQ scores were corrected by combining the first two response-scales for each question: “not experienced at all” and “present but no more of a problem”; i.e., a score of 1 was reduced to 0 (32). These corrected RPQ-16 scores were used in all further analyses.

Total corrected RPQ-16 scores ≥16 were used as an outcome marker of significant post-concussion symptoms, as previously suggested (33).

#### 2.2.2. Functional impairments

After reviewing the many available questionnaires designed to assess function after TBI (34), an adapted and abbreviated questionnaire suitable for remote self-reporting was used, such that it could be completed in a short period of time in the application. The leading question and list of functional impairments enquired about were “have you had trouble with any of the following?”: “difficulty driving or cycling?,” “difficulty doing paperwork?,” “difficulty remembering appointments?,” “difficulty with mental maths?,” “difficulty with conversations?,” “difficulty planning ahead?,” “other difficulty?” or “None”. Participants could choose one or more functional deficits listed (binarized present/absent) (Table 2).

Cramer’s V was used to evaluate correlations between functional deficit categories.

### 2.4. Hierarchical clustering

PCA and CFA are the most commonly used methods to cluster post-concussive symptoms (9). The reason hierarchical clustering was used instead, is that PCA attempts to identify composite symptoms to explain the variances in RPQ-16 symptom severities, while CFA only tests prior hypotheses on underlying latent factors that could “predict” the data (35). Both PCA and CFA can be problematic when applied to ordinal variables such as the RPQ, because both methods assume variables are linear functions of components/factors (36). Thus, when these methods are applied to the RPQ-16, they assume that symptom severities can be approximated as interval data, which may not always be the case (9). For example, it is unclear whether “moderate” symptoms of irritability are equivalent to “moderate” symptoms of double vision, or whether the interval from “present but no more of a problem” to “mild” is the same as the interval between “moderate” to “severe.” For these reasons, agglomerative hierarchical clustering was used (37).

Agglomerative hierarchical clustering has the advantage that, unlike CFA, it is unsupervised and not biased by the current clinical thinking about the latent structures of post-concussion symptoms, and unlike PCA, does not preserve global variance at the potential cost of losing local similarities (26). Additionally, agglomerative hierarchical clustering on spearman rank correlations does not assume a linear relationship between symptom severities and underlying latent components.

As post-concussion symptoms may change within the first year (23), and persistent symptoms are more likely to be associated with functional deficits (15), the main focus of this study was the clustering of symptoms, and their functional deficits, in the prolonged group.

First, the pairwise Spearman rank correlation matrix between the RPQ-16 symptom severity scores were obtained. Next, the clustermap function from seaborn (version 0.11.1) was used to perform unsupervised agglomerative hierarchical clustering on the correlation matrix using the default Euclidean distance metric. The obtained symptom-clusters were compared with those of somatic, emotional, and cognitive symptoms obtained using previous methods (11,21).

In agglomerative hierarchical clustering, individual symptoms are paired first (bottom-up) and a symptom-cluster was defined to be formed when all 16 symptoms of the RPQ were paired with at least one other symptom in as few hierarchical levels as possible. In turn, superclusters were defined similarly as the hierarchical clustering of symptom-clusters together (37).

### 2.5. Analysis of symptom-clusters and functional impairments

Theoretical constructs of questionnaires are only useful insofar as they help with clinically relevant stratification (38). Therefore, in addition to the hierarchical structure of the RPQ-16, functional impairments were also analyzed.

#### 2.5.1. By symptom-cluster membership

The association between functional deficits and symptom-clusters in the prolonged group was investigated. This analysis *by symptom-cluster membership* categorically assigned each individual to none or more symptom-cluster(s). Membership of a symptom-cluster was defined by requiring a participant’s mean corrected RPQ scores for each symptom-cluster to be 1) at least mild or 2) at least moderate (32). For each of these levels, separate Bonferroni corrections were performed at alpha 0.05 (five clusters, six functional impairments, alpha = 0.05/30 = 0.0017). Two-by-two chi-squared tests (or Fisher’s exact where appropriate) were used to investigate the proportion of reported functional impairments.

#### 2.5.2. By functional impairment

To determine if functional impairments were generally associated with more severe symptoms irrespective of symptom-clusters, the mean RPQ-16 severity scores between individuals with no functional impairment (“none”) and those with any reported functional impairment were compared using a Mann-Whitney U test.

To determine if the severity of reported symptom-clusters varied between given functional deficits, all participants that reported a functional deficit (i.e., excluding “none”) were selected, and Mann-Whitney U tests compared the mean scores for each symptom-cluster. This analysis *by functional impairment* was a categorical assignment to one or more functional impairment group(s) with a summary mean severity score for each symptom-cluster. In addition to symptom-clusters, the overall RPQ-16 severity scores for each functional deficit was analyzed. The analyses were Bonferroni corrected for multiple comparisons at alpha 0.05 (five symptom-clusters and one overall RPQ-16, six functional impairments, alpha = 0.05/6 × 6 = 0.0014).

Note that in both analyses described above, by *symptom-cluster membership* (categorical-categorical) and by *functional impairments* (categorical-ordinal), participants may have belonged to more than one symptom-cluster or more than one functional deficit group, respectively.

### 2.6. Sensitivity analyses

In the above *by symptom-cluster membership* analysis, symptom-cluster membership was defined as *mean corrected scores* at least 1) mild or 2) moderate. Sensitivity analyses were performed for the associations that were significant after Bonferroni correction by changing the criteria for inclusion in a symptom-cluster from “mean score” to either “any single symptom” or “all symptoms” from the symptom-cluster (see **Supplementary Methods** for further details).

All analyses and figures were produced using python: pandas v 1.1.5, scipy v 1.5.2, matplotlib v 3.3.3, and seaborn v 0.11.1 (39).

### 2.7. Comparison of RPQ-16 in early vs prolonged groups

In order to better understand the data from this cohort and compare it to previous cohorts, the severity of all reported RPQ-16 symptoms between the two groups were compared using univariate two-by-two chi-squared or Fisher’s exact test as appropriate.

### 2.8. Data availability

Anonymized analysis-ready data and the statistical analyses are available upon reasonable request at the repository (please contact us to gain access): https://github.com/letsmindstep/ClusteringRPQ

## 3. Results

### 3.1. Participants and demographics

2235 people downloaded the application and consented to data collection, of which 523 reported having had at least one concussion, while 1712 responded as not having had a diagnosed concussion. From the 523 participants with at least one concussion, 467 (89.3%) completed the RPQ-16, of whom 22 reported a concussion within the previous 12 months (early), and 445 reported a concussion twelve months or more previously (prolonged).

Of the 445 with prolonged symptoms, 153 (34.4%) had significant post-concussion symptoms (total RPQ-16 score of 16 or more). 10 (45.5%) of 22 participants in the early group had significant symptoms (RPQ-16 score of 16 or more).

There was no significant difference in the age distributions between the early (mean 44.9 years ± 17.4) or prolonged cohorts (47.2 years ± 12.9; Welch’s t-test *p* = 0.54). Demographics, comorbidities, and numbers of past concussions for the prolonged group are shown in **Table 1** and for the early group in **Supplementary Table 1**.

### 3.2. Agglomerative hierarchical clustering

**Figure 1.**
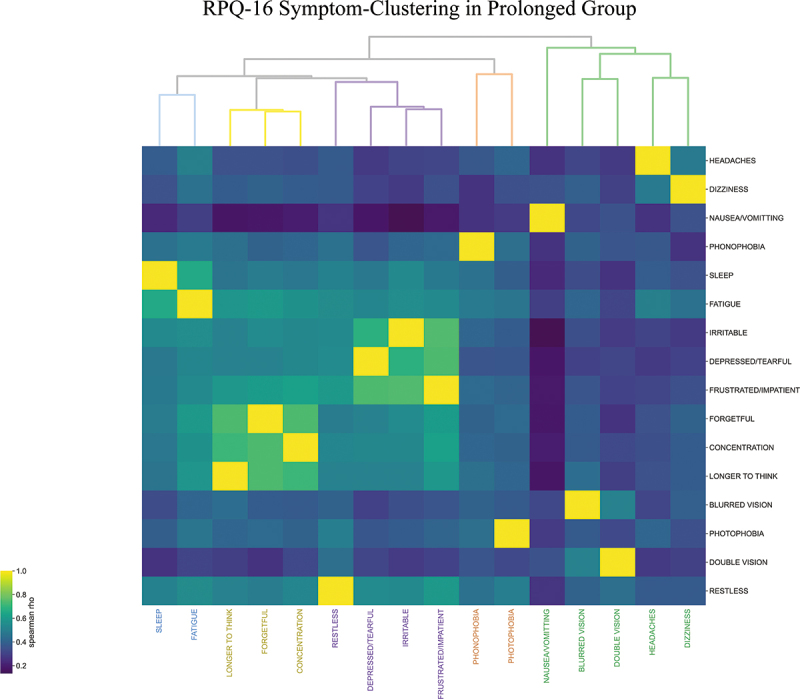
Agglomerative hierarchical clustering of RPQ-16 symptoms 12 months after concussion (prolonged group, n = 445).

Clustering for prolonged symptoms demonstrated five clinical clusters (Figure 1). The *headache-cluster*included blurred vision and double vision in addition to the RPQ-3 (headache, dizziness, nausea and/or vomiting).

Somatic symptoms of sensitivity to light and sound constituted the*sensitivity-cluster*.

A *sleep-fatigue-cluster, cognitive-cluster* (difficulty concentrating, feeling forgetful and taking longer to think); and a *mood-cluster* (restlessness, depression, irritability and frustration) completed the RPQ-16 symptoms in five clusters. As the height of the dendrogram signifies the difference between clusters, these latter three clusters constituted a supercluster with the most similarity (Figure 1).

These five clusters represent similarities between the RPQ questions as assessed by similarities in severity perceived by participants, not as intended by the questionnaire or interpreted by the physician. The clusters and their constituent symptoms were obtained unsupervised. The height of each node in the dendrogram in Figure 1 is inversely proportional to the similarity between its constituent symptoms. Therefore, the *headache-related*cluster (in green) and *sensitivity-cluster*(brown) were associated with the most variance amongst their symptoms, while the three cognitive symptoms (yellow) were the most similar. As migraines are most likely responsible for the symptoms reported in the headache-related and sensitivity clusters, one interpretation of this is that while the severity of the various symptoms that constitute migraines were variable, cognitive symptoms such as forgetfulness, taking longer to think, and lack of concentration were amongst the most semantically equivalent RPQ-16 symptoms.

The Spearman rank correlation matrices for both early and prolonged groups, which were used for hierarchical clustering, are shown in **Supplementary Figure 1**.

Hierarchical clustering in the early group (**Supplementary Figure 2**) was different from that of the prolonged group, however this is considered preliminary due to only 22 participants.

### 3.3. Comparison of RPQ-16 in early vs prolonged groups

For baseline comparisons between these groups, see **Supplementary Table 2** and **Supplementary Results**.

### 3.4. Functional impairments

From amongst the 467 total participantswho had a concussion and completed the RPQ, 254 individuals’ self-reported functional deficits were available (54.4%), of which 240 were from the prolonged group.

The most common responses were difficulty with conversations (44.9%), completing paperwork (37%) and remembering appointments (34.3%, Table 2). The least common functional impairment reported by only 3.9% was “other deficit(s).” This small percentage of unspecified “other deficit(s),” along with previous results that showed these functional impairments were associated with cognitive measures (28), suggest that the functional deficits experienced after a concussion were adequately captured by the smartphone application. As “other” deficits were nonspecific and only reported by 10 individuals, this was omitted from further analyses.

**Figure 2.**
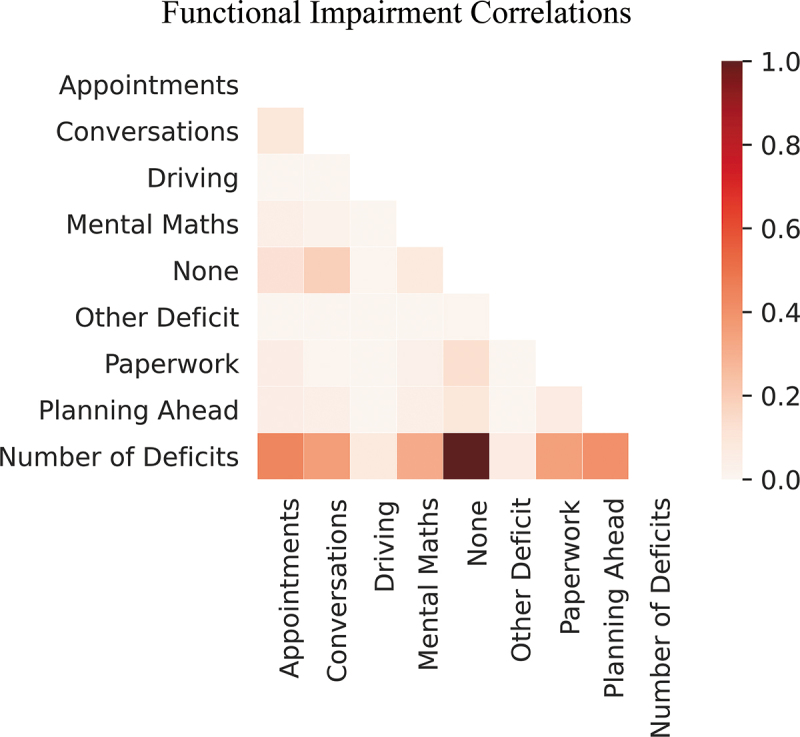
Cramer’s V categorical correlations for functional impairments. Number of deficits was the sum of the number of reported deficits from this list (excluding “None”).

Due to multiple correlations on Cramer’s V, the “number of deficits” was removed from further analyses (Figure 2).

### 3.5. Analysis of symptom-clusters and functional impairments

In the prolonged group, the association between symptom-clusters (Figure 1) and functional impairments were investigated from two perspectives: by symptom-cluster membership (categorical-categorical) and by functional impairments (categorical-ordinal).

### 3.5.1. By symptom-cluster membership

The results of the at-least-mild severity thresholds are shown in Table 3 and the results of the moderate-to-severe symptoms are shown in Table 4.

#### 3.5.1.1. At Least Mild Severity

Results showed that of the self-reported functional impairments, trouble with appointments and conversations were associated with the sleep-fatigue and cognitive clusters, while trouble with conversations was additionally associated with emotional cluster symptoms (Table 3). The largest effect size was for the cognitive-cluster, whereby they were more likely to report difficulties with remembering appointments and following conversations with OR 2.93 (95% CI 1.61–5.34, *p* < 0.001) and OR 4.41 (2.47–7.88, *p* < 0.0001), respectively.

Difficulty driving and trouble with executive planning were not significantly reported more frequently in any of the symptom-clusters after correction for multiple comparisons (Table 3).

Difficulty with mental arithmetic was reported more frequently in the sensitivity to light and sound cluster with OR 2.98 (1.63–5.45, *p* < 0.001), and sleep-fatigue cluster with OR 2.64 (1.46–4.78, *p* < 0.001).

Reports of impairments at filling out paperwork were significantly more frequent in the sleep-fatigue (OR 2.79 [1.61–4.82], *p* < 0.001) and emotional clusters (OR 2.57 [1.49–4.44], *p* < 0.001).

Sensitivity analyses, where the membership criteria for symptoms-clusters were changed from mean symptoms to at-least one symptom or all-symptoms satisfying mild severity, showed the findings were robust except for the association between the sleep-fatigue cluster and mental arithmetic on one out of two of the sensitivity tests. Having at least one mild symptom resulted in an insignificant odds ratio (Table 3).

In summary, the fewest Bonferroni corrected functional impairments were associated with headache (none) and sensitivity-clusters (one), while the greatest number of functional impairments was associated with the sleep-fatigue-cluster (four). Therefore, of all the symptom-clusters with at least mild severity, the sleep-fatigue cluster was associated with the greatest number of functional impairments. This result was robust to sensitivity analyses and whether results were corrected for multiple comparisons or not (Table 3).

#### 3.5.1.2. Moderate-to-severe

A threshold of moderately severe symptoms resulted in both the greatest number of functional impairments and the largest effect sizes to be associated with the cognitive cluster. Participants who reported moderate-or-severe symptoms from the cognitive-cluster (longer to think, difficulty concentrating, being more forgetful) also reported significantly more impairments (five) compared to the sleep-fatigue-cluster (two) and the emotional-cluster (three). The strength of these associations can be found in Table 4.

From 10 significant symptom-cluster/functional-impairment associations, seven were robust to both sensitivity analyses, while three from the emotional symptom-cluster were no longer significant when all symptoms from the cluster were moderately severe (Table 4).

#### 3.5.2. By functional impairment

Individuals who reported any of the functional deficits generally had RPQ-16 severity scores 14.2 points higher than those who reported none (mean RPQ-16 score with any impairment 21.2 vs mean without any impairment 7.0, MWU statistic = 68931.5, *p* < 0.0001).

After correction for multiple comparisons, there were only three significant results for the analysis by functional impairment. Participants who reported difficulty with remembering appointments or following conversations had more severe RPQ-16 total symptoms compared to those who did not report these impairments (Table 5). Having trouble following conversations was associated with more severe RPQ-16 symptoms (mean score with impairment 23.9 vs 17.8 without impairment, *p* < 0.0001), and difficulty remembering appointments was also associated with more severe symptoms (mean score with impairment 23.7 vs 19.3 without impairment, *p* < 0.001). Specifically, those who reported difficulty following conversations also reported more severe cognitive cluster symptoms which included forgetfulness, difficulty concentrating, and taking longer to think (mean cluster score 6.2 with impairment vs 4.4 without impairment,*p* < 0.0001) (Table 5).

## 4. Discussion

### 4.1. Overview

This study is the first to use agglomerative hierarchical clustering on post-concussion symptoms as measured by the RPQ. The five unsupervised symptom-clusters obtained from 445 individuals’ responses to the RPQ-16 at least one year after a concussion were: *headache-related, sensitivity* to light and sound, *sleep-fatigue, cognitive*, and *mood-related*.

Of all the symptom-clusters with at least mild severity, the sleep-fatigue cluster was associated with the greatest number of functional impairments, suggesting that mild symptoms of sleep and fatigue may be more disabling than even mild cognitive symptoms. Therefore, even mild trouble sleeping and mild fatigue should be clinical priorities to screen for and treat. However, with increasing severity of symptoms including at least moderate symptoms, the cognitive cluster was the most functionally disabling. The cognitive cluster included symptoms of taking longer to think, forgetfulness, and difficulty concentrating; and at moderate or more severe severities addressing these symptoms should also be prioritized.

These results also established that the presence of any functional impairment was associated with more severe RPQ-16 symptoms, with an average score 14.2 points higher on the RPQ-16 scale than those who reported none.

### 4.2. Hierarchical RPQ-16 structure

The RPQ-16 questionnaire captures the severity of symptoms after TBI, and a recent review evaluated all the studies that investigated its underlying latent structure using PCA and CFA (9). PCA and CFA often give similar results (20). For example, PCA methods have suggested three components in a study on 200 individuals with TBI: mood and cognition, general somatic, and visual somatic (22); while CFA methods have suggested three similar underlying factors: somatic, emotional, and cognitive (11,21).

This study's hierarchical clustering results are most consistent with this three-factor model: **Cognitive**: forgetfulness, poor concentration, taking longer to think. **Emotional**: irritable, depressed or tearful, frustrated, restless. **Somatic**: headache, dizziness, nausea or vomiting, sensitivity to noise or sound, sleep disturbance, fatigue, blurred vision and double vision (21). The major difference is that instead of sleep-fatigue being within a “somatic” group, hierarchical clustering suggested that they are a separate cluster, and, for at-least mild severities, associated with the most functional impairments. A further difference is that sensitivity symptoms were also clustered separately.

The results of applying PCA and CFA to RPQ-16 have not been homogenous, with some identifying four factors instead of three, e.g., in 181 military veterans (4), and some suggesting dynamic changes in factor structures within the first year after TBI (23), while others have contradicted this (24). Another study assumed six symptomatic RPQ-16 groups during the early post-TBI stage, including “cognitive-fatigue”, “vestibular”, “oculomotor”, “anxiety/mood”, “migraine”, and “cervical”. However, this particular study used a modified RPQ-16, including symptoms that do not feature in the standard RPQ-16 (40). Such studies assume homogeneity of interpretation of symptom descriptions between the clinician and patients completing the questionnaire. The unsupervised clustering method used in this study does not make this assumption. Furthermore, they only profiled early TBI symptoms, during which symptom interpretations may vary from those of chronic symptoms.

Some have suggested using the first three (RPQ-3) and last 13 questions (RPQ-13) as separate measures for early and prolonged symptoms, respectively (41). The RPQ-3 contains questions from the headache cluster, and as these were common, it is unsurprising that RPQ-3 and RPQ-13 have been shown to be good constructs for people with head injury with good retest reliability and external construct validity (41). In other words, hierarchical clustering suggests RPQ-3 may be a special subset of the headache symptom-cluster. As preexisting comorbidities, including migraine, may be associated with higher risks of persistent post-concussion symptoms (42), their treatment should be made a clinical priority. The *sensitivity-cluster* straddled between the *headache-cluster* and the supercluster which included *mood* symptoms, suggesting possible similarities between them. This is in line with previous migraine studies that have found bidirectional associations not just between depressive symptoms and migraine (43), but also specifically with photophobia (44).

This study is the first to use agglomerative hierarchical clustering on RPQ-16. This clustering method groups symptoms based on how well their severity co-varies with all other symptoms on the RPQ-16. While there may not be a strong correlation between two symptom severities, they may still be clustered together due to a strong correlation of their two-by-two spearman correlations with all other symptoms (similarity). This overcomes the incongruence between physicians and patients in the interpretation of symptoms by using a patient-reported measure that is not dependent on the provider’s interpretation of terminologies to co-cluster similar symptoms, as is the case with CFA. Therefore, hierarchical clustering is a more analytically sound approach to define post-concussion RPQ-16 symptom-clusters (45).

### 4.3. Symptom-clusters may stratify functional impairments

Studies have shown no significant differences between RPQ-16 scores in patients with normal or abnormal MRI brain imaging, whether evaluated with a binary threshold (>19 or >35) or using non-parametric tests (46). This highlights the importance of our study in directly evaluating the relationship between clinical symptom-clusters and functional impairments.

From all five symptom-clusters with at least mild severity, sleep-fatigue was associated with the greatest number of functional impairments, highlighting the role mild-to-severe sleep disturbance and fatigue can have in functional impairments. Conversely, moderate-to-severe cognitive symptoms were more functionally disabling than other symptoms. These results therefore suggest that mild cognitive symptoms may be associated with fewer disabling impairments than mild sleep disturbances or fatigue, while more severe cognitive symptoms may be associated with more functional impairments. Symptom-clustering may therefore have clinical value in stratifying functional impairments post-TBI.

Previous studies investigating the association between RPQ scores and outcomes have used the Glasgow Outcome Scale Extended (GOSE), finding that 27.1% (198/731) of participants at 6-months had post-concussion functional impairment when using a GOSE cutoff score less than or equal to 6 (21).It is acknowledged that dichotomization of GOSE can reduce sensitivity (34), therefore, this study used individual functional impairments without an overall functional score. Their study found 6 or more symptoms from the RPQ-16 with a score of at least moderate severity was associated with the highest percentage of dichotomized functional impairments, more than use of the three-factor model. Six or more at-least moderate symptoms from amongst the RPQ-16 is however arbitrary, and their analysis investigating RPQ functional outcomes is categorical-categorical; our current study, however, used both categorical-categorical and the underlying ordinal data to ascertain the presence of correlations – the latter more in line with recommendations (47). Additionally, they assigned individuals to the three-factor model if *any* of the items of the factors had at least moderate severity, whereas our study used the mean of *all* the items of a cluster as more representative of an RPQ-cluster, in addition to further sensitivity analyses. Nevertheless, despite their use of different cutoffs for the RPQ and use of the GOSE questionnaire, their functional impairment results are in line with this study’s results which found that more severe RPQ symptoms are associated with more functional impairments (21).

The list of functional impairments used in this study mostly relate to deficits in attention, memory, or executive function, including difficulty tracking conversations, completing paperwork, remembering appointments, and planning ahead (Table 2). These activities require constant attention, which has previously been shown to objectively be correlated with self-reported cognitive symptoms (48). This explains why moderate-to-severe cognitive symptoms were found in this study to be associated with nearly all of the functional impairments (Table 4). Dysexecutive symptoms have also previously been shown to be correlated with cognitive and emotional symptoms (48), similar to the associations found here between symptom-clusters and functional impairments, where increasing severity of symptoms in the cognitive and emotional clusters were associated with more functional impairments (Table 4 **vs** Table 3).

### 4.4. Sleep-fatigue cluster: potential clinical target?

Although the RPQ does not differentiate between specific sleep complaints, minor TBIs are known to contribute to insomnia, specifically delayed sleep phase and irregular sleep-wake patterns (49). The prevalence of insomnia one year after a concussion is three times the rate found in the general population (50).

Sleep is known to have a neuroprotective role, and its disturbance is associated with mood disturbances, fatigue, and cognitive impairments (51,52), similar to the supercluster and findings in this study that associate them with functional impairments. There is potentially a single pathophysiological mechanism underlying this post-concussion symptom-supercluster, that of the functional disruption of the network between the cortex and dentate nucleus of the hippocampus (53). This results in dysregulated slow-wave sleep and trouble retrieving memories (52,53), which is the archetypal cognitive symptom at the center of the cognitive-cluster (“forgetfulness” in Figure 1).

Screening for sleep difficulties could aide recovery (50), and a recent study has reviewed pharmacological and psychological interventions for sleep disturbances after TBI (54). In fact, reconsolidation of the 24-hour sleep-wake cycle after TBI has been purported to predict emergence from posttraumatic amnesia as well as cognitive impairment in rehabilitation settings (55).

The data and results from this study are therefore important in shaping understanding of symptomatology and potential recovery in those who have notably prolonged symptoms for over 12 months.

The sleep-fatigue cluster results should be taken in the context of the spectrum of TBI and symptom severities. Results suggest that at least mild sleep and fatigue disturbances after mild TBI are associated with significant impairments in daily function, even more than cognitive symptoms. However, at the mild end of TBI, prior studies have shown that difficulty sleeping in 346 individuals was associated with worse post-concussive symptoms including cognition (50). At the moderate-to-severe TBI spectrum in hospitalized patients, previous studies have also shown that sleep quality was linearly associated with consciousness and cognitive function (55). While they measured sleep quality objectively using continuous wrist actigraphy and measures of sleep-wake consolidation, sleep duration and sleep fragmentation index; the RPQ-16 utilized in this present hierarchical clustering study used subjective single scale measures for sleep, fatigue and the three cognitive symptoms. It would therefore be ideal in future to link the subjective RPQ-16 symptoms of difficulty sleeping and cognitive difficulties with objective measures of sleep quality and cognition, to assess the degree of correlation between subjective and objective measures.

### 5.5. Limitations

Although the value of the term “concussion” is disputed by some authors as it has no verifiable ground-truth (56), and is heterogeneously defined by some to be as mild as “any blow to the head” (11), nevertheless the question used in the application mentioned “diagnosed concussion” to identify suitable participants. This is likely to signify symptomatic mild-TBI as per guidelines (57), and is more suited for use in the remote collection of self-reported symptoms as it is easier to understand. Additionally, although self-reported data may be susceptible to selection and recall biases, a prior study using this same application’s data has demonstrated epidemiological validity (28).

Nearly a quarter of the users of the application from which data was collected reported having had a diagnosed concussion (523/2235). As the Mindstep application (Mindset4Dementia) was designed as a screening tool for cognitive impairment, the sample therefore comprised of individuals with mild TBI who were worried about cognitive repercussions, for example due to prolonged symptoms, and may not be representative of all TBI cases with prolonged symptoms. Nevertheless, this study replicated some previous results, indirectly validating our participant cohort (see **Supplementary Resul**t**s** comparing the two early and prolonged groups).

RPQ-16 asks about relative symptoms compared to before a concussion, but it is conceivable that some participants would report the absolute severity of their current symptoms, compounded by the high prevalence of RPQ-16 symptoms in individuals without TBI (17,58). Symptoms developed and reported as post-concussive may not have been conclusively caused by a concussion, as the concussion could have been incidental, and coincidental comorbidities could result in similar symptoms (12). Therefore, symptoms may be incorrectly attributed to the concussion, however, this is a known limitation in all RPQ studies (59). As a result, the symptom-clusters identified in all RPQ studies, irrespective of methodology, may not be specific to post-concussion syndrome.

Although a bespoke list of functional impairments were carefully curated, this list has not been previously validated. Nevertheless, there was an “other deficit(s)” category which was the least selected at 3.9%, suggesting this list of impairments captured most functional impairments.

Another point to highlight is that a cutoff of 12 months was used to define more prolonged symptoms, whereas persistent post-concussion syndrome is usually defined as over 3 months (10). 12.8% of individuals with prolonged symptoms in this study reported having had COVID-19 (Table 1), which may impact the reporting of symptoms.

This study was observational so causality cannot be inferred, only a prospective design can establish whether the identified symptom-clusters may be causally associated with the development of specific functional impairments. Further investigation of sleep-related symptoms would require use of objective sleep measures. Future studies assessing post-concussion symptoms should also ask about preexisting comorbidities such as migraines and sleep disturbances prior to TBI.

## 6. Conclusions

The strength of hierarchical clustering, unlike previous methods, is that results are not biased by how clinicians intended for the questions to be interpreted and may be especially useful in self-administered questionnaires on subjective experiences of symptoms. Using this method, our findings suggest we should rethink RPQ-16 symptoms in individuals 12 months after a concussion as having five symptom-clusters, where the severity of symptom-clusters are associated with functional impairments. Specifically, at least mild difficulty sleeping and fatigue, and moderate-to-severe cognitive symptoms, were more functionally disabling than other similarly rated symptoms.

Our results are important in shaping understanding of symptomatology and recovery from concussion in those who have notably prolonged symptoms and may help with rehabilitation strategies targeting sleep and fatigue. Our results suggest that focused clinical screening should be performed routinely, and are supportive of the increased involvement of neurologists in the care of patients with concussion to help diagnose and treat associated underdiagnosed and undertreated conditions, as previously proposed (56).

Further studies are required to replicate our findings using agglomerative hierarchical clustering in other cohorts and questionnaires, establish the causal direction of sleep and fatigue problems with functional impairments, and the effects of intervention.
